# *Aloe vera*/*Chondrus crispus* Hydrocolloid Emulgels as Polymer-Structured Colloidal Carriers for Cannabidiol Incorporation

**DOI:** 10.3390/polym18161988

**Published:** 2026-08-15

**Authors:** Claudia C. Polanía, Luis Alfonso Trujillo-Cayado, Jenifer Santos

**Affiliations:** 1 Departamento de Ingeniería Química, Facultad de Química, Universidad de Sevilla, c/Profesor García-González, s/n, 41011 Sevilla, Spain; clacabpol@alum.us.es; 2 Departamento de Ciencias de la Salud y Biomedicina, Facultad de Ciencias de la Salud, Universidad Loyola Andalucía, Avenida de las Universidades s/n, 41704 Dos Hermanas, Spain

**Keywords:** carrageenan, insect-derived emulsifiers, avocado lipid phase, multiple light scattering, shear-thinning systems, phytocannabinoid delivery, semisolid vehicles, HPLC-UV quantification

## Abstract

Cannabidiol (CBD) is a lipophilic phytocannabinoid of interest for dermocosmetic and topical applications, but its poor aqueous solubility and sensitivity to environmental conditions make its formulation challenging. This work aimed to develop avocado oil-in-water emulgels for CBD incorporation using a mixed emulsifying system based on Kolliphor EL and cricket protein and a commercial *Aloe vera*/*Chondrus crispus* gel as the structuring phase. First, different Kolliphor EL:cricket protein ratios were evaluated according to droplet size distribution. No significant differences in droplet size were found among formulations containing at least 67% Kolliphor EL (*p* > 0.05); therefore, 67K-33G was selected because it maximized the substitution of Kolliphor EL with cricket protein without significantly affecting droplet size. Progressive replacement of water by the commercial hydrocolloid gel markedly increased consistency and viscoelasticity, producing a transition from fluid emulsions to elastic emulgels between 50 AV and 75 AV. The 75 AV formulation showed low Turbiscan Stability Index values, a predominantly elastic response, and a more balanced structure than 100 AV. The selected emulgel showed a total analytical recovery of 96.2 ± 1.2% for CBD and retained 97.3 ± 0.7% of the initially recovered CBD after 30 days of storage at 4 °C protected from light. These results support the potential of *Aloe vera*/*Chondrus crispus*-based emulgels as semisolid carriers for lipophilic bioactive compounds.

## 1. Introduction

Oil-in-water emulsions serve as prevalent colloidal platforms for incorporating lipophilic ingredients into formulations related to cosmetics, pharmaceuticals, and food [1]. The utility of these systems stems from their ability to dissolve poorly water-soluble compounds within the oil phase, which can then be dispersed in an aqueous continuous medium. Nevertheless, these systems are thermodynamically unstable, and their long-term efficacy is contingent upon factors such as droplet size, interfacial stabilization, the viscosity of the continuous phase, and the equilibrium among destabilization mechanisms, including creaming, flocculation, coalescence, and Ostwald ripening [2].

In the context of topical and dermocosmetic applications, emulsions are required to maintain physical stability while also demonstrating appropriate consistency, spreadability, and residence time on the skin [3]. Emulgels are especially appealing in this context due to their capacity to incorporate lipophilic active ingredients, characteristic of emulsions, while also exhibiting the rheological properties typical of gels structured with polymers or hydrocolloids [4]. The gelled continuous phase can decrease droplet mobility and the frequency of droplet collisions. Additionally, shear-thinning behavior aids in the spreading of the product during application and supports structural recovery during storage. *Aloe vera* is extensively utilized in topical formulations due to its inner leaf gel, which comprises water, polysaccharides, minerals, amino acids, vitamins, and phenolic compounds [5]. Notably, high-molecular-weight polysaccharides, such as acetylated glucomannans, are frequently linked to its moisturizing, film-forming, and skin-conditioning properties. Nevertheless, the technological role of a commercial *Aloe vera* gel cannot always be interpreted as that of fresh Aloe parenchymatous gel alone, since marketed gels may contain additional hydrocolloids, antioxidants, and preservatives to ensure adequate consistency and shelf life.

From a formulation point of view, *Aloe vera* and *Chondrus crispus* can be considered complementary natural ingredients for the development of topical emulgels. *Aloe vera* gel is widely appreciated in cosmetic and dermopharmaceutical formulations due to its high water content and the presence of bioactive compounds, particularly polysaccharides such as acetylated glucomannans, which have been associated with moisturizing, film-forming, and skin-conditioning properties [6]. However, the rheological contribution of *Aloe vera* alone is usually moderate and may depend strongly on its botanical origin, processing conditions, and commercial formulation [7]. For this reason, commercial *Aloe vera* gels often include additional hydrocolloids that help provide the consistency, stability, and sensory properties required for topical application. In the commercial gel used in this study, *Aloe vera* is combined with *Chondrus crispus*, a red seaweed known as a source of sulfated polysaccharides, mainly carrageenan-type compounds [8]. These marine hydrocolloids are able to bind water and form structured continuous phases through thickening and gel-forming mechanisms [9,10]. Therefore, *Chondrus crispus* should not be considered merely as a minor additive, but rather as a relevant technological component of the system. Its presence may reinforce the aqueous hydrocolloid network, increase viscoelasticity, and reduce the mobility of the dispersed oil droplets.

Within this structured continuous phase, the emulsifying system also plays a central role in defining droplet formation and interfacial stabilization. Kolliphor EL, a non-ionic surfactant, can rapidly reduce interfacial tension and facilitate droplet breakup during high-energy homogenization [11]. Its partial replacement by cricket protein introduces an alternative proteinaceous ingredient with potential interfacial activity and aligns with the current interest in sustainable and unconventional protein sources [12,13]. The integration of a low-molecular-weight surfactant with a protein can result in the formation of a mixed interfacial layer surrounding the droplets. However, the ultimate size and distribution of the droplets are contingent upon the relative proportions of these components and the specific conditions of homogenization.

Cannabidiol (CBD) is a non-intoxicating phytocannabinoid of interest for topical formulations due to reported antioxidant, anti-inflammatory, sebostatic, and skin-conditioning effects [14]. Its practical formulation is challenging because CBD is highly lipophilic, has very low aqueous solubility, and may undergo degradation depending on light, temperature, oxygen exposure, and matrix composition [15]. Lipid-based colloidal systems and emulgels are considered effective carriers for CBD, as they facilitate the solubilization of the active compound in a compatible oil phase and its dispersion within a structured aqueous vehicle [16]. Cannabinoids, beyond their technological significance as bioactive compounds, have garnered significant attention due to their extensive pharmacological potential. Their interaction with the endocannabinoid system may affect various physiological processes, such as immune regulation, inflammatory responses, pain perception, and redox homeostasis. Notably, CBD is a non-psychoactive cannabinoid linked to potential anti-inflammatory, antioxidant, and immunomodulatory activities. However, these effects are contingent upon the dose, route of administration, and biological context. Further research is necessary to determine their efficacy and safety for specific applications [17].

This study aimed to formulate an oil-in-water emulgel for the incorporation of CBD, utilizing avocado oil as the lipid carrier, Kolliphor EL and cricket protein as the emulsifying system, and a commercial *Aloe vera*/*Chondrus crispus* gel as the natural structuring agent. Initially, different Kolliphor EL:cricket protein ratios were screened based on droplet size distribution to select a suitable mixed emulsifying system. Subsequently, the impact of incrementally substituting water with the commercial *Aloe vera* gel on rheology and physical stability was evaluated. Finally, CBD incorporation and short-term storage stability were assessed using HPLC-UV. Unlike our previous study, which used cricket flour as the sole emulsifier and rhamsan gum as a thickener [13], the present work combines purified cricket protein with Kolliphor EL, uses an *Aloe vera*/*Chondrus crispus* gel as the structuring phase, and evaluates the resulting emulgel as a vehicle for CBD incorporation and short-term retention.

## 2. Materials and Methods

### 2.1. Materials

Organic avocado oil (Chosen Foods, San Diego, CA, USA) served as the dispersed lipid phase. The aqueous phase was formulated with distilled water, Kolliphor EL (Merck-Sigma-Aldrich, Darmstadt, Germany) as a non-ionic surfactant, and cricket protein (99% purity; Thailand Unique, Udon Thani, Thailand) as a protein-based emulsifying agent. Commercial *Aloe vera* gel (99% purity; B.O.T., Málaga, Spain) functioned as a natural polymer-rich rheological modifier and was employed as a partial or complete substitute for the aqueous phase. Cannabidiol (CBD; CAS 13956-29-1; purity > 98%) was procured from Merck-Sigma-Aldrich (Darmstadt, Germany). For chromatographic analysis, HPLC-grade methanol, ultrapure water, and 0.22 µm PTFE syringe filters were utilized.

Avocado oil was selected as the dispersed lipid phase due to its suitability for topical formulations, its emollient characteristics, and its capacity to solubilize lipophilic compounds such as CBD. Kolliphor EL, a high-HLB non-ionic surfactant, was chosen for its effectiveness in forming oil-in-water emulsions. Furthermore, cricket protein was investigated as a partial protein-based substitute with potential interfacial activity. The commercial *Aloe vera*/*Chondrus crispus* gel was utilized as a polymer-rich structuring phase. In this system, the *Aloe vera*-based medium provides skin-conditioning and film-forming components, while the seaweed-derived polysaccharides from *C. crispus* contribute to continuous-phase thickening and gel formation. Thus, this combination was considered suitable for developing a structured, spreadable vehicle for the topical incorporation of CBD.

### 2.2. Preparation of Emulsions and Emulgels

Oil-in-water emulsions were prepared in 40 g batches. The base formulation comprised 20 wt.% avocado oil and 2.5 wt.% total emulsifying system, with the aqueous phase constituting the remaining 77.5 wt.% necessary to complete the formulation. The oil and total emulsifier contents were predetermined as fixed formulation parameters, based on prior findings with cricket-based avocado-oil emulsions [13]. These concentrations were not independently optimized in this study; rather, they were held constant to isolate the effect of the relative Kolliphor EL:cricket protein proportion and subsequently assess the substitution of water with the commercial *Aloe vera*/*Chondrus crispus* gel. A theoretical HLB calculation was not applied to the mixed emulsifying system, as proteins lack a defined HLB value and the required HLB of avocado oil may vary with its composition. Consequently, the Kolliphor EL:cricket protein ratio was experimentally screened while maintaining constant oil and total emulsifier contents. The initial evaluation included the following Kolliphor EL:cricket protein ratios: 100:0, 75:25, 50:50, 25:75, and 0:100. Based on preliminary droplet-size results, an additional formulation with a 67:33 ratio was prepared and utilized for subsequent emulgel development.

The continuous phase was formulated at room temperature (approximately 25 ± 2 °C) by dispersing specified quantities of Kolliphor EL and cricket protein in distilled water. The mixture underwent ultrasonic treatment for up to 15 min at room temperature, without external heating or thermostatic regulation. While a minor temperature increase may have occurred during sonication, no cloudiness, phase separation, or localized thickening was detected. Consequently, a visually homogeneous continuous phase was achieved prior to emulsification.

A two-stage emulsification process was utilized due to the complementary effects of rotor-stator and probe-sonication treatments. Initially, the Ultra-Turrax treatment produced a homogeneous coarse pre-emulsion by dispersing the oil phase within the continuous phase and reducing the initial droplet size. Subsequently, probe sonication facilitated further droplet disruption through acoustic cavitation, resulting in a finer and more uniform emulsion. During probe sonication, the sample was kept in an ice bath to minimize heat accumulation. Primary homogenization was carried out using an Ultra-Turrax T25 rotor-stator homogenizer (IKA, Staufen, Germany) at 9500 rpm for 60 s. A secondary homogenization step was then performed using an ultrasonic processor (VCX750/VCX500, Sonics & Materials, Newtown, CT, USA) equipped with an acoustic enclosure. Sonication was applied at 45% amplitude for 17.5 min under pulsed conditions (5 s on/5 s off). The samples were kept in an ice bath during sonication to minimize temperature increases.

For the *Aloe vera*-containing systems, the selected 67:33 Kolliphor EL:cricket protein ratio was fixed and the aqueous phase was progressively replaced by *Aloe vera* gel. Five formulations were prepared: 0 AV, 25 AV, 50 AV, 75 AV, and 100 AV, corresponding to 0, 25, 50, 75, and 100% replacement of the aqueous phase by *Aloe vera* gel, respectively. In all cases, the avocado oil concentration was kept at 20 wt.% and the total emulsifying system at 2.5 wt.%.

### 2.3. Cannabidiol Loading

CBD was incorporated into the formulation by dissolving it in the avocado oil phase prior to emulsification, leveraging its lipophilic properties. A final concentration of 0.10 wt.% CBD was chosen, equating to 40 mg of CBD per 40 g batch. The CBD was accurately weighed in an amber glass vial, combined with avocado oil, and subjected to magnetic stirring at 40 ± 2 °C for 15 min. The mixture was subsequently treated in an ultrasonic bath for 5 min to accelerate dissolution and ensure complete homogenization. This procedure was applied consistently to all three independently prepared batches before emulsification. The CBD-infused oil phase was then cooled to room temperature before proceeding with emulsification. CBD-loaded emulgels were prepared in triplicate and stored in sealed amber glass containers at 4 °C until analysis. A non-emulsified avocado oil sample with the same CBD concentration was prepared under identical conditions and served as a control.

### 2.4. Droplet Size Distribution

The droplet size distributions were assessed through laser diffraction employing a Mastersizer 2000 instrument (Malvern Instruments, Malvern, UK). Distilled water served as both the dispersing medium and the blank. Samples were incrementally introduced into the stirred dispersion unit until an appropriate optical concentration was achieved, thereby reducing multiple scattering effects. The size distribution was characterized by the Sauter mean diameter (D_3,2_) and the span parameter.

### 2.5. Physical Stability by Multiple Light Scattering

Physical stability was evaluated using a Turbiscan Lab analyzer (Formulaction, Toulouse, France). Freshly prepared samples were placed in cylindrical glass vials and analyzed without dilution. The instrument scans the sample height using near-infrared light (approximately 880 nm) and records transmission and backscattering signals using detectors positioned at 0° and 135°, respectively. Backscattering profiles were monitored along the vial height to detect destabilization phenomena such as creaming, sedimentation, flocculation, or coalescence. The Turbiscan Stability Index (TSI) was calculated for both the bottom region and the whole sample, allowing comparison of the global destabilization intensity among formulations. Approximately 25 mL of each freshly prepared sample was placed in a cylindrical glass vial and analysed without dilution at 25 °C for 30 days. Backscattering and transmission profiles were recorded at 1 h intervals over the entire sample height. The Turbiscan Stability Index (TSI) was calculated for both the bottom region and the whole sample. 

### 2.6. Rheological Characterization

Rheological measurements were conducted using a Haake MARS 40 rheometer (Thermo Fisher Scientific, Karlsruhe, Germany) at 20 ± 0.1 °C. Before measurement, the samples were allowed to equilibrate in the measuring system for 120 s. No solvent trap or equivalent evaporation-protection system was used, and no visible sample drying was observed during the tests. Initial tests were undertaken to determine the suitable measuring geometry. The optimized low-viscosity emulsion was assessed using a CC25 coaxial cylinder geometry, whereas the *Aloe vera*/*Chondrus crispus*-containing emulgels were analyzed with 60 mm roughened parallel-plate geometry with a gap of 1 mm to minimize wall slip. Steady-state flow curves were obtained over a shear-rate range of 0.1–30 s^−1^, using an increasing shear-rate ramp on a logarithmic scale in ascending order, with a measurement time of 1 min at each point. The experimental data were fitted to the Power-law model:

$$\eta =k\cdot {\dot{\gamma }}^{n-1}$$

where *η* is the viscosity, *k* is the consistency index and *n* is the flow index (*n* < 1 denotes shear-thinning behavior). The most structured formulations were also fitted to the Herschel–Bulkley model to evaluate the possible contribution of an apparent yield stress. As this model did not provide an appreciable improvement in the coefficient of determination, the Power-law model was retained for all formulations to ensure a consistent comparison of the fitting parameters.

Oscillatory stress sweeps were performed at 1 Hz over a stress range of 0.1–100 Pa. The LVR was defined as the stress interval within which G′ remained within ±5% of its plateau value. Frequency sweeps were subsequently conducted from 3 to 0.3 Hz at a constant stress of 0.1 Pa for each formulation, selected within the corresponding LVR. Storage modulus (G′), loss modulus (G″), and loss tangent (tan δ) were recorded as functions of frequency. All rheological measurements were performed in duplicate.

### 2.7. HPLC-UV Determination of CBD and Total Analytical Recovery

CBD was quantified by reversed-phase HPLC-UV using a method adapted from Morakul et al. [18]. The chromatographic separation was carried out using a C18 column (250 × 4.6 mm, 5 µm). The mobile phase consisted of methanol:water (85:15, *v*/*v*) under isocratic conditions at a flow rate of 1.2 mL/min. The injection volume was 20 µL, the detection wavelength was 220 nm, the column temperature was 25 °C, and the total run time was approximately 12 min. A CBD stock solution (1.0 mg/mL) was prepared in methanol and diluted to obtain calibration standards between 1.25 and 40 µg/mL. Each standard was injected in triplicate, and linearity was considered acceptable when the coefficient of determination was at least 0.99. A partial validation of the adapted method was performed in the 75 AV emulgel matrix. Selectivity was assessed by analysing a blank 75 AV emulgel without CBD and verifying the absence of an appreciable interfering signal at the CBD retention time. Accuracy and repeatability were evaluated by spiking blank 75 AV samples with CBD before extraction at three concentration levels, with each level analysed in triplicate. Accuracy was expressed as percentage recovery and repeatability as relative standard deviation (RSD). Linearity was evaluated over the calibration range of 1.25–40 µg/mL.

For CBD extraction, 0.100 g of emulgel was accurately weighed into a 10 mL volumetric flask and brought to volume with methanol. The sample was vortexed for 1 min, sonicated for 15 min to disrupt the emulsion structure and promote CBD extraction, and centrifuged at 10,000× *g* for 10 min. The supernatant was filtered through a 0.22 µm PTFE syringe filter and transferred to amber HPLC vials. Blank emulsions prepared without CBD were analyzed to verify the absence of interfering peaks from avocado oil, Kolliphor EL, cricket protein, or *Aloe vera* gel. The total analytical recovery (TAR) of CBD was calculated as:

$$TAR\left(\%\right)=\frac{C_{exp}}{C_{theo}}\cdot 100$$

where *C_exp_* is the experimentally determined CBD concentration and *C_theo_* is the batch-specific theoretical CBD concentration, calculated from the actual mass of CBD added and the final mass of the corresponding emulgel batch. Although the target nominal concentration was 1.0 mg/g, the gravimetrically determined *C_theo_* values used in the calculations were 0.984, 0.992, and 1.022 mg/g for batches 1–3, respectively.

### 2.8. Statistical Analysis

Measurements were conducted in triplicate or more, unless stated otherwise, with results presented as mean ± standard deviation. Statistical differences among the formulations were assessed using one-way analysis of variance (ANOVA), followed by Tukey’s test. A *p*-value of less than 0.05 was considered indicative of statistical significance.

## 3. Results and Discussion

### 3.1. Selection of the Kolliphor EL:Cricket Protein Ratio

**Figure 1:**
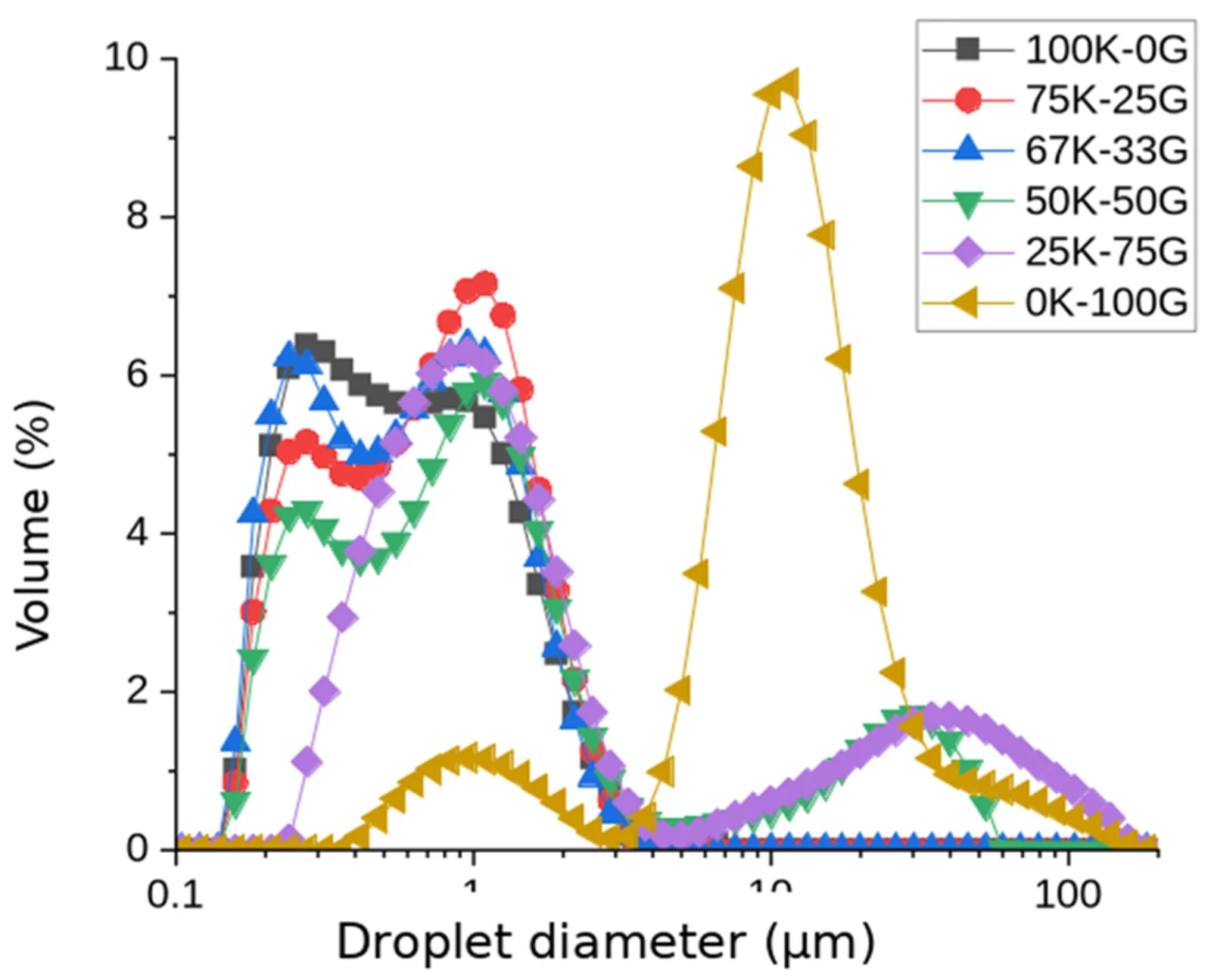
Droplet size distributions of the emulsions prepared with different Kolliphor EL (K) and cricket protein (G) ratios.

The first formulation stage evaluated the effect of the relative proportion of Kolliphor EL (K) and cricket protein (G) on the droplet size distribution of avocado oil-in-water emulsions. Figure 1 shows the droplet size distributions obtained for the six systems, while Table 1 summarizes the Sauter mean diameter (D_3,2_) and span values. The results demonstrate that the balance between the non-ionic surfactant and the protein-based ingredient strongly affected both droplet disruption during homogenization and the final degree of polydispersity.

Emulsions with a substantial amount of Kolliphor EL, specifically the formulations 100K-0G, 75K-25G, and 67K-33G, demonstrated the smallest droplet sizes, with D_3,2_ values between 424 and 475 nm. Statistical analysis indicated no significant differences among these formulations (*p* > 0.05), suggesting that substituting up to one-third of the surfactant with cricket protein does not hinder the formulation’s ability to produce submicrometric droplets under the given processing conditions. The 67K-33G emulsion exhibited the lowest D_3,2_ value (424 ± 30 nm), closely matching the fully Kolliphor-stabilized emulsion (427 ± 32 nm). This outcome is technologically significant, as it implies that the protein component can partially replace the conventional non-ionic surfactant while maintaining a similar initial droplet size.

The behavior observed in Kolliphor EL-rich formulations can be understood by examining the distinct molecular characteristics and adsorption dynamics of the two emulsifying components. Cricket proteins possess both hydrophobic and hydrophilic amino acid residues, endowing them with the amphiphilic properties necessary for adsorption at oil–water interfaces. Upon reaching the interface, these proteins may undergo partial conformational rearrangement, aligning hydrophobic regions toward the avocado oil phase while maintaining hydrophilic regions in the aqueous phase. This adsorption process can decrease interfacial tension and form a viscoelastic protein-containing layer that offers steric and, depending on the protein charge, electrostatic resistance against droplet coalescence. Aprile et al. [12] experimentally demonstrated the adsorption of cricket protein isolates at oil–water interfaces, noting a significant reduction in interfacial tension, with sigmoidal adsorption isotherms similar to those of conventional animal proteins. However, protein adsorption is generally slower than that of low-molecular-weight surfactants due to the involvement of molecular diffusion, interfacial attachment, and subsequent conformational rearrangement. Consequently, Kolliphor EL can more rapidly reach and cover newly generated interfaces during high-energy homogenization, facilitating droplet disruption and limiting immediate recoalescence. At moderate cricket protein proportions, both components may contribute to a heterogeneous mixed interfacial layer: Kolliphor EL provides rapid initial coverage, while the protein adsorbs at remaining interfacial regions and reinforces the layer post-rearrangement. This combination may elucidate why replacing up to one-third of Kolliphor EL did not significantly increase droplet size.

Nonetheless, the interaction should not be presumed to be exclusively synergistic. Low-molecular-weight non-ionic surfactants and proteins can compete for available interfacial area, and surfactant adsorption may hinder protein attachment or partially displace previously adsorbed protein molecules [19]. As the total emulsifier concentration remained constant, increasing the cricket protein fraction simultaneously reduced the amount of rapidly adsorbing Kolliphor EL. At intermediate and high protein proportions, slower adsorption, limited protein dispersibility, and potential aggregation may have impeded sufficiently rapid coverage of all newly formed droplets. This would favor recoalescence and account for the broader and more heterogeneous distributions of the 50K-50G and 25K-75G systems. Since interfacial composition, adsorption kinetics, and protein conformational changes were not directly measured, this mixed-layer mechanism should be considered a plausible interpretation of the droplet-size results rather than direct experimental evidence.

Increasing the relative proportion of cricket protein beyond a certain range resulted in larger and more heterogeneous systems. The formulations 50K-50G and 25K-75G exhibited significantly higher D_3,2_ values and notably broader distributions. This pattern was consistent with the emergence of secondary coarse populations in the droplet size curves, as illustrated in Figure 1. The exceptionally high span values of the 50K-50G and 25K-75G formulations (21.724 and 38.877, respectively) are consistent with the markedly heterogeneous and multimodal distributions observed in Figure 1. Since span is defined as (D_90_ − D_10_)/D_50_, these values indicate a very wide separation between the distribution percentiles, arising from the coexistence of very fine droplets and a pronounced coarse-droplet population. This suggests that part of the oil phase was efficiently disrupted, whereas another fraction underwent insufficient disruption or recoalescence because of incomplete or insufficiently rapid interfacial coverage. These findings indicate that at elevated protein fractions, the interfacial coverage and/or adsorption kinetics were inadequate to stabilize all newly formed oil-water interfaces during homogenization. The emulsion stabilized solely with cricket protein demonstrated the highest mean diameter (4774 ± 229 nm), confirming that cricket protein alone was insufficient to produce a fine emulsion under the specified preparation conditions. Consequently, the 67K-33G ratio was selected as a practical starting formulation for subsequent emulgel development. This selection was not based on statistical superiority, since its D_3,2_ did not differ significantly from those of 100K-0G and 75K-25G, and 75K-25G exhibited a slightly narrower span. Instead, 67K-33G represented the highest cricket-protein proportion that maintained a small mean droplet size statistically comparable to that obtained with Kolliphor EL alone, thereby allowing one-third of the conventional surfactant to be replaced. Nevertheless, this selection should be regarded as preliminary because rheological behaviour, interfacial properties, economic feasibility, and environmental performance were not compared at this screening stage.

### 3.2. Effect of Aloe vera/Chondrus crispus Gel as a Rheological Modifier

**Figure 2:**
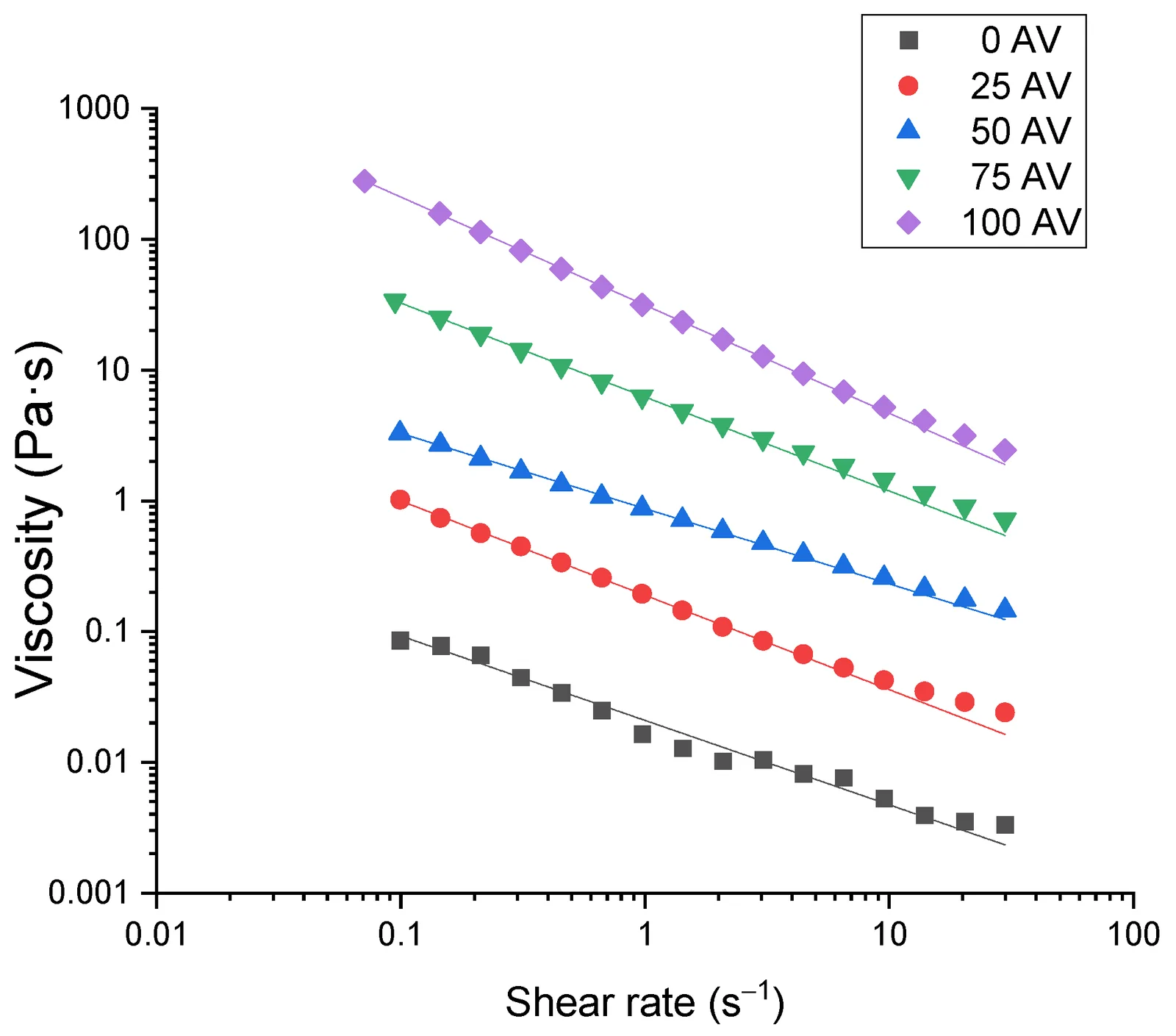
Flow curves of the emulsions/emulgels prepared with increasing *Aloe vera*/*Chondrus crispus* Gel-content. AV is the percentage of the aqueous phase replaced by the *Aloe vera*/*Chondrus crispus* gel. Symbols correspond to experimental data and the solid lines to the power-law fit. Error bars represent the standard deviation but are not visible because they are smaller than the corresponding data symbols.

Following the selection of the 67K-33G emulsifying system, the aqueous phase was systematically substituted with a commercial *Aloe vera*/*Chondrus crispus* gel to assess its efficacy as a natural rheological modifier and structuring agent. The formulations produced were designated as 0 AV, 25 AV, 50 AV, 75 AV, and 100 AV, corresponding to the proportion of the aqueous phase replaced by the commercial hydrocolloid gel. It is crucial to understand that this substitution does not merely involve the incorporation of *Aloe vera* mucilage; rather, it entails the addition of a composite *Aloe vera*/seaweed-derived hydrocolloid phase, wherein *Chondrus crispus* polysaccharides are anticipated to significantly contribute to thickening and gel formation. Although the incorporation of the commercial *Aloe vera*/*Chondrus crispus* gel may also affect the apparent droplet size, this stage focused mainly on the rheological structuring and physical stabilization of the selected 67K-33G emulsion. In highly structured emulgels, droplet size measurements should be interpreted with caution, since hydrocolloid networks or weak droplet aggregates may contribute to the apparent size distribution after dilution. Figure 2 illustrates the impact of this substitution on the flow behavior of the formulations, while Table 2 provides a summary of the parameters derived from the power-law fitting.

All formulations demonstrated shear-thinning behavior, as evidenced by the reduction in apparent viscosity with an increase in shear rate and flow index values below unity. This characteristic is advantageous for topical semisolid formulations, as it combines high viscosity at rest, which enhances physical stability, with ease of spreading under the shear forces generated during application [20]. The consistency index increased markedly with the replacement of water by the commercial *Aloe vera*/*Chondrus crispus* gel.

The polysaccharides in *Aloe vera* may aid in water retention and moderate thickening. However, the significant increase in viscoelasticity and the development of a gel-like network are more likely due to the carrageenan-type sulfated polysaccharides from *Chondrus crispus*, which have a notably higher gelling capacity. As both components were introduced as a premixed commercial gel and not evaluated independently, their specific contributions cannot be experimentally quantified. Consequently, the observed effects should be ascribed to the composite hydrocolloid phase, with *Chondrus crispus* probably providing the main structural contribution. Such rheological structuring is typically anticipated when polymeric or biopolymeric phases enhance the viscosity of the continuous medium and limit droplet mobility [21]. Although the structured emulgels may exhibit an apparent yield-stress-like response, fitting their flow curves to the Herschel–Bulkley model did not result in an appreciable improvement in R^2^ compared with the Power-law model. Consequently, the latter was applied consistently to the entire formulation series, enabling a direct comparison of the consistency and flow behaviour indices.

The increase in viscosity was not proportional to the amount of commercial gel incorporated. Although 25 AV and 50 AV showed higher viscosity than 0 AV, the most pronounced increase was observed when the replacement level reached 75 AV and 100 AV. This non-linear behavior suggests that a critical concentration of hydrocolloid material is required to form a sufficiently connected continuous-phase network. Below this concentration, the hydrocolloid molecules mainly increase the viscosity of the aqueous phase, whereas at higher concentrations they may interact more effectively and generate a stronger three-dimensional structure. This behavior is consistent with the transition from a fluid emulsion to a true emulgel. The flow index did not vary monotonically with increasing hydrocolloid content, ranging from 0.28 to 0.43. This behaviour indicates that the degree of shear-thinning was not governed exclusively by the amount of commercial gel, but also by the microstructure developed at each replacement level and its reorganization under shear. In particular, the comparatively higher n value of 50 AV suggests a less pronounced shear dependence than that of 25 AV and 75 AV, despite its higher consistency index. Moreover, k and n are correlated empirical parameters of the power-law fit and should therefore be interpreted jointly rather than independently. Nevertheless, all n values remained well below unity, confirming the shear-thinning behaviour of every formulation.

**Figure 3:**
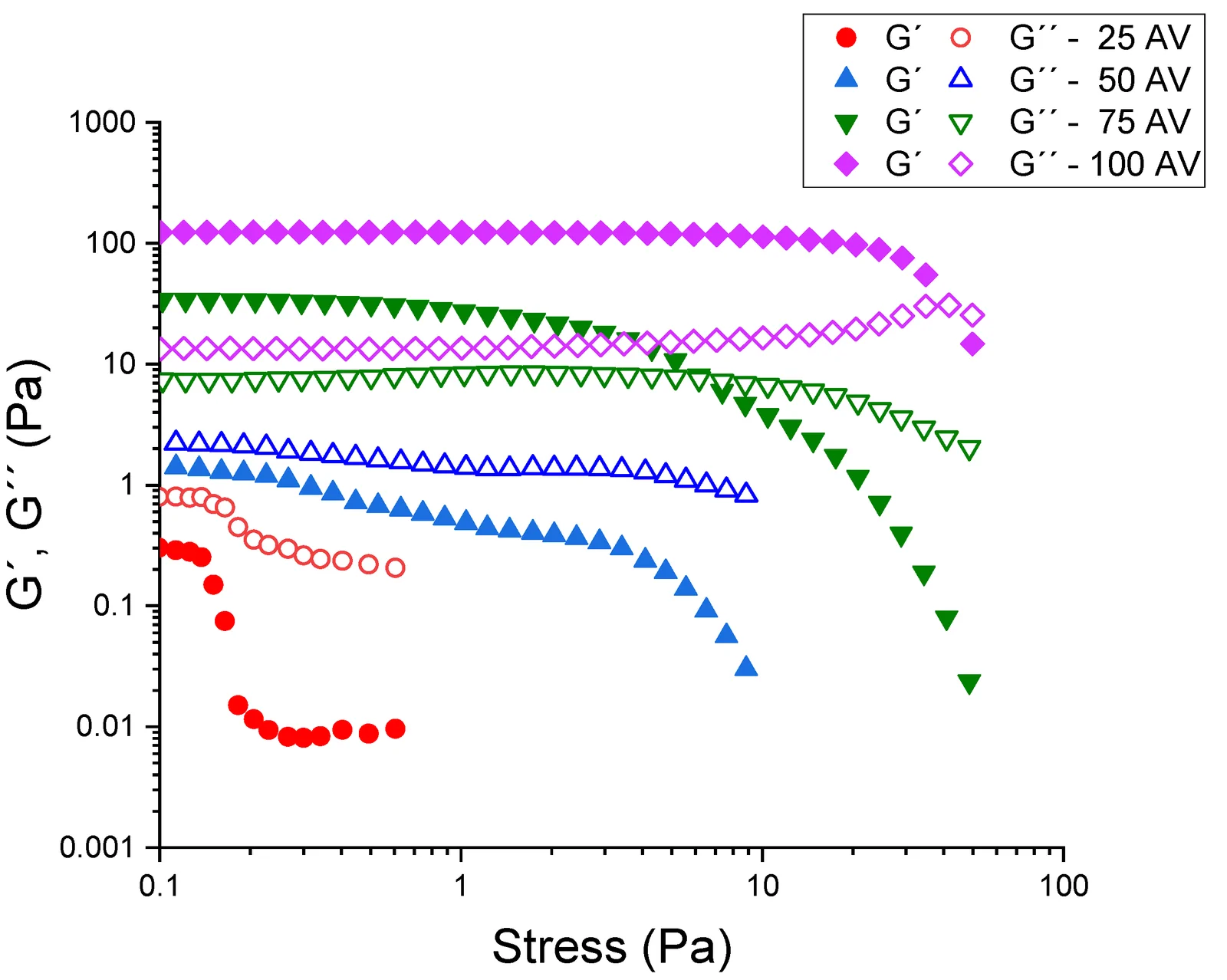
Stress sweep tests of the *Aloe vera*/*Chondrus crispus* Gel-containing formulations. Error bars represent the standard deviation but are not visible because they are smaller than the corresponding data symbols.

Oscillatory measurements further substantiated the progressive transition from a fluid emulsion to a structured emulgel. As illustrated in Figure 3, the stress sweep tests demonstrated distinct variations in the mechanical strength of the formulations. The 25 AV sample exhibited characteristics of a weakly structured viscoelastic system, characterized by low storage modulus (G′) and loss modulus (G″) values, along with a limited linear viscoelastic region. An increase in the commercial gel content to 50 AV resulted in elevated values for both moduli, indicating a higher degree of internal structuring. Nonetheless, this formulation continued to exhibit a significant viscous contribution, implying that the continuous phase had not yet developed a fully elastic network.

A gel-like response was distinctly observed for both 75 AV and 100 AV formulations. In these systems, the storage modulus (G′) exceeded the loss modulus (G″) within the low-stress region, indicating a predominance of the elastic component over the viscous one. This behavior is typical of emulgels, where dispersed oil droplets are embedded within a structured hydrocolloid network. The 100 AV formulation exhibited the highest viscoelastic moduli and the greatest resistance to applied stress, confirming the formation of a robust internal structure. However, from the perspective of topical application, excessive structuring may be disadvantageous as it can reduce spreadability and complicate handling, pumping, or application. Consequently, the 75 AV formulation offers a more balanced composition, featuring a well-defined elastic network but with lower consistency compared to 100 AV. Table 3 illustrates that augmenting the *Aloe vera*/*Chondrus crispus* gel content progressively enhanced the internal structure of the formulations. The 25 AV and 50 AV systems displayed low plateau moduli, with G_LVR_″ exceeding G_LVR_′, indicating a predominantly viscous nature. Conversely, the 75 AV and 100 AV systems exhibited G_LVR_′ surpassing G_LVR_″, signifying the formation of an elastic gel-like network. The critical stress increased from approximately 0.80 Pa for 75 AV to 15 Pa for 100 AV, alongside a higher crossover stress for 100 AV, indicating its significantly greater resistance to structural breakdown. Collectively, these findings confirm that the primary rheological transition occurred between 50 AV and 75 AV, with 100 AV developing the most robust and stress-resistant network.

**Figure 4:**
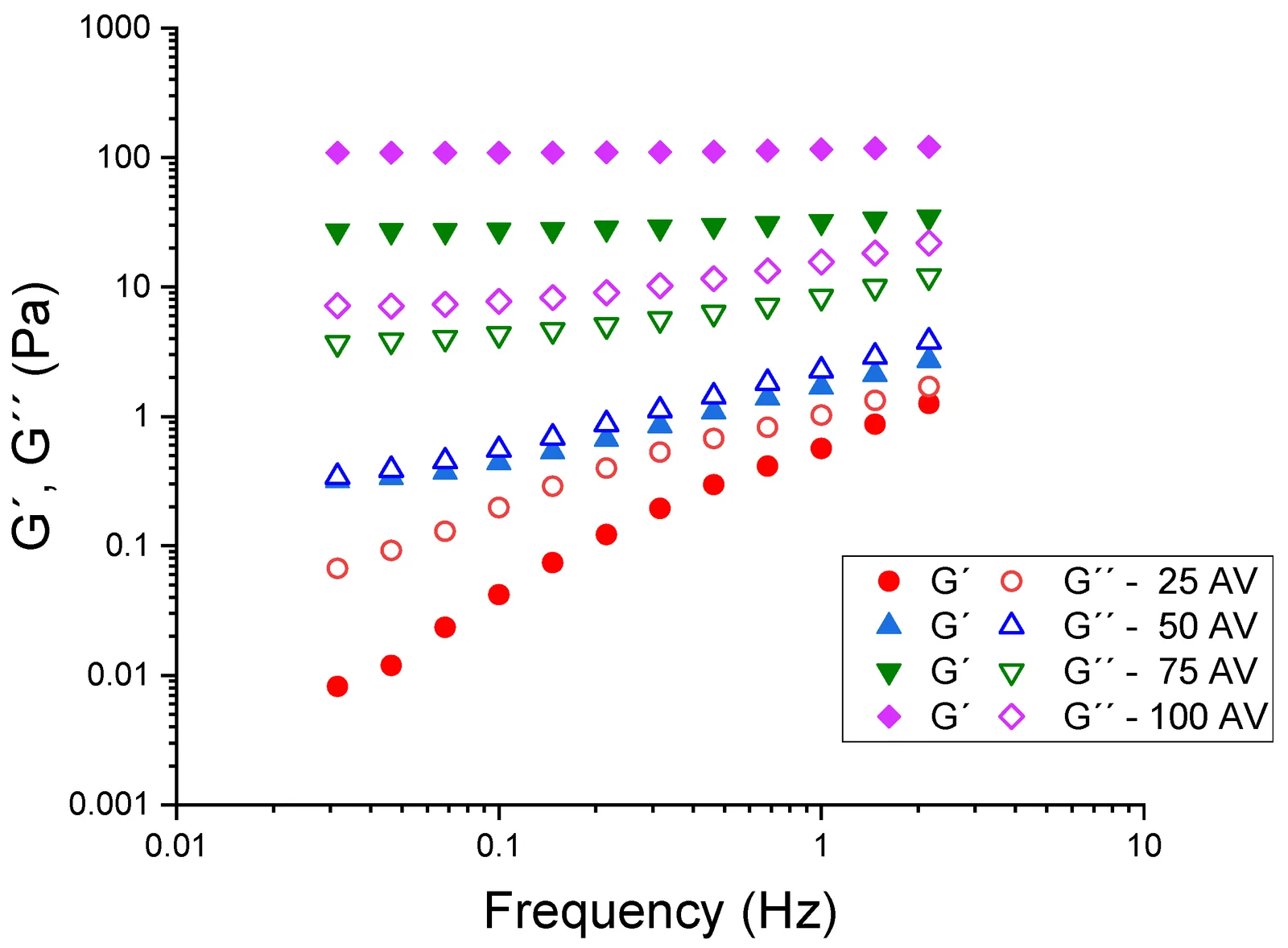
Frequency sweep tests of the *Aloe vera*/*Chondrus crispus* Gel-containing formulations. Error bars represent the standard deviation but are not visible because they are smaller than the corresponding data symbols.

Results from the frequency sweep (Figure 4) corroborated this trend. In the 25 AV formulation, the loss modulus (G″) exceeded the storage modulus (G′) across most of the frequency spectrum, signifying a predominantly liquid-like viscoelastic behavior. The 50 AV formulation exhibited an intermediate response, with both moduli converging at higher frequencies. Conversely, the 75 AV and 100 AV formulations consistently demonstrated G′ values surpassing G″ throughout the frequency range, with minimal frequency dependence. This behavior is characteristic of weak gel-like systems, where the internal structure is sufficiently stable to store mechanical energy over the experimental time scale.

Quantitatively, 75 AV exhibited tan δ values of approximately 0.13–0.33 over the investigated frequency range, whereas lower values, approximately 0.06–0.18, were obtained for 100 AV. Furthermore, fitting the frequency dependence of the storage modulus to G′ = A·ω^n^ yielded low exponents of approximately 0.07 and 0.03 for 75 AV and 100 AV, respectively. These exponents were below 0.2 and, together with tan δ < 1, quantitatively support the weak gel-like classification of both formulations. The lower exponent and loss tangent of 100 AV indicate a more elastic and less frequency-dependent network. Consequently, the primary rheological transition from a viscous emulsion to an elastic emulgel occurred between the 50 AV and 75 AV formulations.

**Figure 5:**
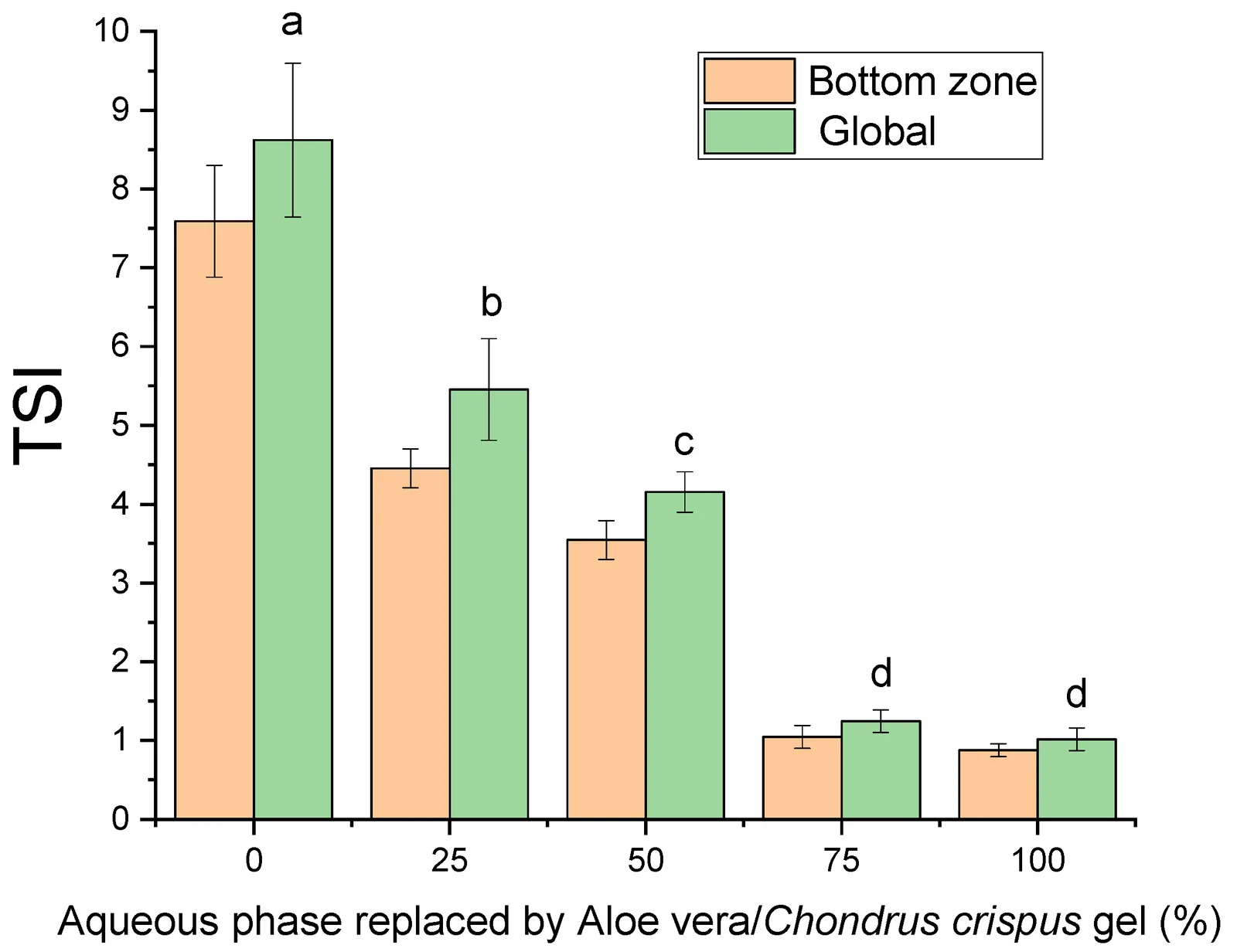
Turbiscan Stability Index (TSI) values after 30 days of storage at 25 °C calculated in the bottom zone and for the total sample as a function of *Aloe vera*/*Chondrus crispus* content. Data are presented as mean values of three replicate measurements. Error bars represent the standard deviation but are not visible because they are smaller than the corresponding data symbols. Different letters indicate statistically significant differences among formulations (*p* < 0.05); formulations sharing the same letter are not significantly different (*p* > 0.05).

The physical stability of the formulations was assessed over 30 days using the Turbiscan Stability Index (TSI), calculated for both the bottom zone of the vial and the entire sample (Figure 5). The values shown correspond to the final measurement at day 30. The reduction in TSI values following incorporation of the *Aloe vera*/*Chondrus crispus* gel indicates lower overall changes in the backscattering profiles during storage, consistent with reduced macroscopic destabilization. The 0 AV emulsion exhibited the highest TSI values, particularly in the lower region, indicating droplet migration and gravitational separation. Conversely, 75 AV and 100 AV demonstrated the lowest TSI values, implying that the hydrocolloid network effectively restricted droplet movement. The observed lower TSI values align with a reduction in macroscopic droplet migration and gravitational separation due to continuous-phase structuring. Nevertheless, these values do not confirm the absence of flocculation or coalescence. Continuous phases enriched with hydrocolloids may facilitate polymer-mediated droplet interactions or form weak flocculated structures, which are indistinguishable from continuous-phase structuring based on the current rheological and multiple-light-scattering data. As microscopy and droplet-size measurements were not conducted on the emulgels before and after storage, the possibility of droplet aggregation or coalescence cannot be ruled out. Although 100 AV displayed the highest viscosity and viscoelastic moduli, its physical stability was not significantly superior to that of 75 AV. Thus, the complete substitution of water with the commercial gel did not confer a distinct technological advantage. The 75 AV formulation was identified as the most suitable emulgel for CBD incorporation, as it exhibited a predominantly elastic response, pronounced shear-thinning behavior, low TSI values, and an optimal balance between physical stability and anticipated ease of topical application.

### 3.3. Cannabidiol Incorporation and Storage Stability

CBD was integrated into the chosen 75 AV emulgel by dissolving it within the avocado oil phase prior to emulsification. This approach was adopted due to CBD’s pronounced lipophilic properties, which facilitate its solubilization in lipid matrices and eliminate the necessity for substantial quantities of organic co-solvents. The nominal concentration was established at 1.0 mg of CBD per gram of formulation, equating to 0.10 wt.%. The partial validation showed adequate analytical performance in the 75 AV emulgel matrix. The calibration response was linear over the studied range (R^2^ ≥ 0.995). Recoveries across the three fortification levels ranged from 91 to 109%, with RSD values below 6% at each level. Moreover, no appreciable interfering signal was observed at the CBD retention time in the blank 75 AV formulation. These results support the suitability of the adapted HPLC-UV method for CBD quantification in the studied formulation under the analytical conditions employed. HPLC-UV analysis revealed a distinct CBD peak at approximately 8.6 min, with no significant signal observed at the same retention time in the blank formulation. This outcome suggests that the primary formulation components, including avocado oil, Kolliphor EL, cricket protein, and the commercial *Aloe vera*/*Chondrus crispus* gel, did not cause notable chromatographic interference under the applied conditions. The retention time was also consistent with previously documented reversed-phase HPLC-UV methods for CBD determination utilizing C18 columns and methanol/water mobile phases [22,23].

The mean total analytical recovery of CBD was determined to be 96.2 ± 1.2%, indicating that most of the CBD initially added to the formulation was recovered after emulgel preparation and sample extraction (Table 4). This high recovery rate is primarily attributed to the prior dissolution of CBD in avocado oil, facilitating the integration of the active compound into a uniform lipid phase before droplet formation. The mixed emulsifying system and the structured continuous phase did not appear to adversely affect CBD recovery during processing. The analytical procedure measured the total CBD recovered from the formulation but did not differentiate between CBD located within the oil droplets, associated with the interfacial region, or potentially solubilized in other colloidal domains. To accurately determine the non-incorporated CBD fraction and calculate encapsulation efficiency in a stricter sense, a specific separation step, such as ultrafiltration or ultracentrifugation, would be necessary. The storage stability of CBD was assessed over 30 days at 4 °C in sealed amber glass containers. The CBD-loaded 75 AV emulgel was compared with a control sample of CBD dissolved in avocado oil. For comparative purposes, the results were normalized to the initial CBD content of each system (Table 5). Both systems maintained a high proportion of CBD during refrigerated storage, consistent with the protective storage conditions employed, including low temperature, absence of direct light exposure, and limited air contact. These conditions are particularly significant as CBD degradation can be accelerated by heat, light, and oxygen exposure.

After 30 days, the 75 AV emulgel retained 97.3 ± 0.7% of its initial CBD content, whereas the avocado oil control retained 95.3 ± 0.6%. The difference between the two systems at day 30 was statistically significant (*p* = 0.021); however, its absolute magnitude was small (2.0 percentage points). Therefore, these results indicate that incorporation into the emulgel did not adversely affect short-term CBD stability, but they are insufficient to demonstrate a protective effect. Moreover, storage was evaluated only for 30 days at 4 °C in sealed amber containers, which represents a mild, protective condition and does not reproduce typical consumer storage or transport conditions. No studies were conducted under ambient or accelerated conditions, and photostability was not assessed.

Nevertheless, this interpretation should be considered as a proposed mechanism rather than as direct experimental proof. Oxygen diffusion, lipid oxidation markers, interfacial composition, and CBD distribution within the different regions of the emulgel were not directly measured in this work. Therefore, the results demonstrate that the selected emulgel allows efficient CBD incorporation and good short-term CBD preservation under refrigerated and light-protected conditions, but further studies would be needed to confirm its protective mechanism. In particular, long-term storage assays, photostability tests, release studies, skin permeation experiments, and a more comprehensive analytical validation, including intermediate precision and robustness, would be required before considering cosmetic or dermopharmaceutical application.

## 4. Conclusions

This study investigated the development of avocado oil-in-water emulgels for the incorporation of CBD, utilizing a mixed emulsifying system comprising Kolliphor EL and cricket protein, with a commercial *Aloe vera*/*Chondrus crispus* gel serving as the structuring phase. Initially, the 67K-33G emulsifying system achieved the smallest Sauter mean diameter, facilitating the partial substitution of the conventional non-ionic surfactant with a protein-based ingredient. Although this formulation did not statistically differ from those containing more Kolliphor EL, it provided an optimal balance between small droplet size, acceptable polydispersity, and ingredient substitution. The replacement of water with the *Aloe vera*/*Chondrus crispus* gel significantly altered the systems’ rheological behavior. All formulations exhibited shear-thinning behavior, with increased gel content enhancing consistency and viscoelasticity. The transition from a predominantly viscous emulsion to an elastic emulgel occurred between 50 AV and 75 AV. The 75 AV and 100 AV formulations exhibited the lowest TSI values, with no significant differences, indicating that complete replacement of water with the gel did not improve stability. Consequently, 75 AV was selected as the most balanced formulation, offering high physical stability, an elastic response, and less structuring than 100 AV, which may be more suitable for topical application. CBD was successfully incorporated into the 75 AV emulgel at 0.10 wt.% by dissolving it in the avocado oil phase. The mean total analytical recovery of CBD was 96.2 ± 1.2%. This value should not be interpreted as encapsulation efficiency because no separation procedure was performed to determine the distribution of CBD among the different formulation domains. After 30 days of refrigerated storage away from light, the emulgel retained 97.3 ± 0.7% of the initial CBD content, slightly exceeding that of the avocado oil control. These findings indicate that incorporation into the *Aloe vera*/*Chondrus crispus*-based emulgel did not adversely affect short-term CBD stability under refrigerated and light-protected conditions. However, the limited duration and mild storage conditions preclude any conclusion regarding a protective effect or stability under consumer storage and transport conditions. The proposed system represents a promising semisolid colloidal vehicle for incorporating lipophilic bioactive compounds such as CBD, combining avocado oil, a mixed surfactant/protein emulsifying system, and a natural hydrocolloid-rich continuous phase. The limitations of this study are noteworthy. The absence of long-term and accelerated stability assessments, along with photostability evaluations, is a significant gap. Additionally, the study did not investigate CBD release or its permeation through the skin. Measurements such as zeta potential, interfacial tension, lipid oxidation markers, and droplet-size distributions were not conducted, nor was in situ microscopy of the structured emulgels performed. This omission prevents the differentiation of droplet-level effects from those due to continuous-phase structuring. Furthermore, the HPLC-UV method was only partially validated, and the interactions between Kolliphor EL and cricket protein at the oil–water interface were not explored. As a result, the proposed mechanisms for stabilization and CBD protection remain preliminary and require further experimental validation. Further research addressing these limitations is required before the suitability of the developed emulgel for cosmetic or dermopharmaceutical applications can be established.

## Figures and Tables

**Table 1:** Sauter mean diameter (D_3,2_) and span of the emulsions prepared with different Kolliphor EL (K) and cricket protein (G) ratios.

Sample	D_3,2_ (nm)	Span
100K-0G	427 ± 32 ^a^	2.437 ± 0.189 ^a^
75K-25G	475 ± 41 ^a^	2.000 ± 0.131 ^b^
67K-33G	424 ± 30 ^a^	2.188 ± 0.151 ^a,b^
50K-50G	580 ± 45 ^b^	21.724 ± 0.879 ^c^
25K-75G	932 ± 85 ^c^	38.877 ± 0.956 ^d^
0K-100G	4774 ± 229 ^d^	2.186 ± 0.189 ^a,b^

Different letters indicate statistically significant differences according to Tukey’s test (*p* < 0.05).

**Table 2:** Power-law fitting parameters for the flow curves as a function of *Aloe vera*/*Chondrus crispus* Gel content. AV, percentage of the aqueous phase replaced by the *Aloe vera*/*Chondrus crispus* gel; k, consistency index; n, flow index; R^2^, coefficient of determination.

Sample	k (Pa·s^n^)	n	R^2^
0 AV	0.0209 ± 0.0014 ^a^	0.36 ± 0.01 ^a^	0.979
25 AV	0.1893 ± 0.0113 ^b^	0.28 ± 0.01 ^b^	0.998
50 AV	0.8711 ± 0.0540 ^c^	0.43 ± 0.01 ^c^	0.999
75 AV	6.2158 ± 0.4182 ^d^	0.28 ± 0.01 ^b^	0.999
100 AV	33.6584 ± 1.5563 ^e^	0.31 ± 0.01 ^e^	0.997

Different letter indicate statistically significant differences according to Tukey’s test (*p* < 0.05).

**Table 3:** Rheological parameters obtained from the stress sweep tests of formulations containing different proportions of *Aloe vera*/*Chondrus crispus* gel. AV, percentage of the aqueous phase replaced by the *Aloe vera*/*Chondrus crispus* gel; G′_LVR_, storage modulus in the linear viscoelastic region; G″_LVR_, loss modulus in the linear viscoelastic region; τ_c_, critical stress; n.d., not determined.

Sample	G′_LVR_ (Pa)	G″_LVR_ (Pa)	τ_c_ (Pa)	G′-G″ Crossover Point (Pa)
0 AV	n.d.	n.d.	n.d.	n.d.
25 AV	0.3 ± <0.1 ^a^	0.8 ± <0.1 ^a^	≈0.15	Not observed
50 AV	1.7 ± 0.1 ^b^	2.2 ± 0.1 ^b^	≈0.25	Not observed
75 AV	36.7 ± 0.8 ^c^	8.1 ± 0.3 ^c^	≈0.80	≈9
100 AV	104.2 ± 2.5 ^d^	13.3 ± 0.5 ^d^	≈15	≈40

Different letters indicate statistically significant differences according to Tukey’s test (*p* < 0.05).

**Table 4:** Initial CBD content and total analytical recovery (TAR) in the 75 AV emulgel. TAR, total analytical recovery; SD, standard deviation.

Batch	Theoretical Concentration (mg/g)	Experimental Concentration (mg/g)	TAR (%)
1	0.984	0.948	96.34
2	0.992	0.966	97.38
3	1.022	0.971	95.01
Mean ± SD	0.999 ± 0.020	0.962 ± 0.012	96.2 ± 1.2

**Table 5:** CBD remaining during storage of the 75 AV emulgel and the avocado oil control. CBD, cannabidiol.

Time (Days)	CBD Retained in Emulgel (%)	CBD Retained in Oil (%)
7	99.2 ± 0.7	98.5 ± 0.8
14	98.1 ± 0.8	96.9 ± 0.4
30	97.3 ± 0.7	95.3 ± 0.6

## Data Availability

The original data presented in the study are openly available at Zenodo: https://zenodo.org/records/21452701 (accessed on 20 July 2026).
